# Dynamic prediction of malignant ventricular arrhythmias using neural networks in patients with an implantable cardioverter-defibrillator

**DOI:** 10.1016/j.ebiom.2023.104937

**Published:** 2023-12-19

**Authors:** Maarten Z.H. Kolk, Samuel Ruipérez-Campillo, Laura Alvarez-Florez, Brototo Deb, Erik J. Bekkers, Cornelis P. Allaart, Anne-Lotte C.J. Van Der Lingen, Paul Clopton, Ivana Išgum, Arthur A.M. Wilde, Reinoud E. Knops, Sanjiv M. Narayan, Fleur V.Y. Tjong

**Affiliations:** a Department of Clinical and Experimental Cardiology, Amsterdam UMC Location University of Amsterdam, Heart Center, Meibergdreef 9, Amsterdam, the Netherlands; b Department of Medicine and Cardiovascular Institute, Stanford University, Stanford, CA, USA; c Department of Biomedical Engineering and Physics, Amsterdam University Medical Center Location University of Amsterdam, Meibergdreef 9, Amsterdam, the Netherlands; d Faculty of Science, University of Amsterdam, Science Park 904, Amsterdam, the Netherlands; e Department of Cardiology, Amsterdam UMC, Location VU Medical Center, De Boelelaan 1118, Amsterdam, the Netherlands; f Department of Radiology and Nuclear Medicine, Amsterdam UMC Location University of Amsterdam, Meibergdreef 9, Amsterdam, the Netherlands; g Department of Information Technology and Electrical Engineering, Swiss Federal Institute of Technology Zurich (ETHz), Gloriastrasse 35, Zurich, Switzerland; h ITACA Institute, Universtitat Politècnica de València, Camino de Vera S/n, Valencia, Spain; i Amsterdam Cardiovascular Sciences, Heart Failure & Arrhythmias, Amsterdam, the Netherlands

**Keywords:** Cardiology, Machine learning, Deep learning, Electrocardiography, Sudden cardiac death

## Abstract

**Background:**

Risk stratification for ventricular arrhythmias currently relies on static measurements that fail to adequately capture dynamic interactions between arrhythmic substrate and triggers over time. We trained and internally validated a dynamic machine learning (ML) model and neural network that extracted features from longitudinally collected electrocardiograms (ECG), and used these to predict the risk of malignant ventricular arrhythmias.

**Methods:**

A multicentre study in patients implanted with an implantable cardioverter-defibrillator (ICD) between 2007 and 2021 in two academic hospitals was performed. Variational autoencoders (VAEs), which combine neural networks with variational inference principles, and can learn patterns and structure in data without explicit labelling, were trained to encode the mean ECG waveforms from the limb leads into 16 variables. Supervised dynamic ML models using these latent ECG representations and clinical baseline information were trained to predict malignant ventricular arrhythmias treated by the ICD. Model performance was evaluated on a hold-out set, using time-dependent receiver operating characteristic (ROC) and calibration curves.

**Findings:**

2942 patients (61.7 ± 13.9 years, 25.5% female) were included, with a total of 32,129 ECG recordings during a mean follow-up of 43.9 ± 35.9 months. The mean time-varying area under the ROC curve for the dynamic model was 0.738 ± 0.07, compared to 0.639 ± 0.03 for a static (i.e. baseline-only model). Feature analyses indicated dynamic changes in latent ECG representations, particularly those affecting the T-wave morphology, were of highest importance for model predictions.

**Interpretation:**

Dynamic ML models and neural networks effectively leverage routinely collected longitudinal ECG recordings for personalised and updated predictions of malignant ventricular arrhythmias, outperforming static models.

**Funding:**

This publication is part of the project DEEP RISK ICD (with project number 452019308) of the research programme Rubicon which is (partly) financed by the 10.13039/501100003246Dutch Research Council (NWO). This research is partly funded by the 10.13039/100019741Amsterdam Cardiovascular Sciences (personal grant F.V.Y.T).

**Research in context**

**Evidence before this study**

A comprehensive search was conducted on June 26th, 2023, using PubMed's title/abstract database. The search terms used were “Ventricular arrhythmia” OR “Sudden cardiac death” OR “ICD shock” AND “Machine Learning” OR “Deep Learning” AND “ECG” OR “electrophysiology.” The search yielded 25 relevant papers. All of these studies used supervised machine learning techniques, specifically for prediction of malignant ventricular arrhythmias using static data. None of these studies addressed a dynamic machine learning model using longitudinal ECG data.

**Added value of this study**

We present the development and validation of a dynamic machine learning model that is capable of providing updated predictions, leveraging electrocardiograms (ECG) recorded over time. Using an autoencoder, a type of neural networks for unsupervised learning, information was extracted from 6-lead ECGs to a latent space and used to predict malignant ventricular arrhythmias treated by an implantable cardioverter-defibrillator. Integrating these longitudinal ECG-derived features within a dynamic machine learning model resulted in improved predictive accuracy (time-varying area under the receiver operating characteristic curve (AUROC) of 0.738 ± 0.07), compared to a static model (time-dependent AUROC of 0.639 ± 0.03). Feature importance analysis and latent space traversal were used to understand and evaluate how different features affect the ECG morphology and model predictions.

**Implications of all the available evidence**

High volumes of personalised electrophysiological data collected over time are effectively leveraged by supervised and unsupervised models to facilitate dynamic predictions of malignant ventricular arrhythmias. The feasibility of translating this approach to clinical practice and generalisability of findings to different patient populations should be investigated in a prospective study.

## Introduction

Ventricular arrhythmias are an important cause of sudden cardiac death (SCD), affecting approximately 250,000 cases in the European Union alone.^1^ The core component of SCD prevention in patients at high risk, is an implantable cardioverter-defibrillator (ICD).^2^, ^3^, ^4^ Risk-stratification for SCD is typically based on static risk predictors, such as a history of sustained ventricular arrhythmias (ventricular tachycardia (VT) or ventricular fibrillation (VF)) and a reduced left ventricular ejection fraction (LVEF) despite optimal medical treatment.^2^ However, the complex and dynamic pathophysiological processes that underlie the onset of malignant ventricular arrhythmias are unlikely to be fully reflected by static measurements and baseline clinical patient characteristics alone.^5^ The vast amounts of personalised electrophysiological data collected real-time through digital health tools, such as wearables and intra-cardiac devices, may reflect dynamic changes in the arrhythmogenic substrate and triggering mechanisms preceding arrhythmia onset.^6^^,^^7^ Machine learning methods (ML) that can accommodate time-varying and baseline data have emerged as an alternative to prognostic models that rely on static data only. In contrast to common statistical techniques, these dynamic ML models can learn non-linear relationships and patterns within multimodal longitudinal dataset to provide updated predictions.^8^, ^9^, ^10^ Therefore, we hypothesised that the high volumes of (ambulatory) ECG recordings collected over time could be leveraged within a dynamic ML framework to predict the risk of impending ventricular arrhythmias.

Furthermore, deep learning models have been established as a superior approach for detection of ECG signatures that are unrecognisable by the human eye and impossible to obtain through classic signal theory/processing techniques hitherto.^8^^,^^11^, ^12^, ^13^, ^14^ State-of-the-art deep learning models have been shown to be effective for detection of arrhythmias and diagnosis of cardiomyopathies, genetic heart diseases, valvular pathologies and prediction of patient outcomes.^8^^,^^15^^,^^16^ Among these techniques, variational autoencoders (VAEs), a class of artificial neural network capable of learning patterns and structures in data without relying on explicit labelling, enable the representation of data to a compressed, latent space. This compressed representation of the ECG summarises the key features of the original signal, and may be used to generate new samples from the encoded variables. Prior studies have demonstrated encoder-decoder architecture neural networks to be able to extract physiologically-relevant ECG features in a latent feature space.^17^, ^18^, ^19^, ^20^, ^21^ Considering that this latent space provides a comprehensive representation of the underlying ECG brought back to a pre-defined number of variables, it can be subsequently used for classification and regressions tasks.^22^ In this study, we examine the potential of accommodating a supervised dynamic ML model with the learned low-dimensional representations from longitudinal ECGs spanning a duration of 44 months. We hypothesised that a ML model that incorporates dynamic time-varying ECG features as well as static clinical data would better predict malignant ventricular arrhythmias, whose pathophysiology is known to result from dynamic as well as static clinical and structural factors.

## Methods

### Study design

A multicentre, retrospective, observational study using patient data obtained from two hospitals in Amsterdam, The Netherlands, was performed (Amsterdam Medical Center and the VU University Medical Center). Patients implanted with an ICD with or without resynchronisation therapy (CRT) between 2007 and 2021 for primary or secondary prevention of SCD were included. Implanted devices were single-chamber ICDs (VR, n = 1076), dual-chamber ICDs (DR, n = 788), subcutaneous ICDs (S-ICD, n = 399) and cardiac resynchronisation therapy-defibrillators (CRT-D, n = 679). The defibrillators used were manufactured by Biotronik (Germany), Medtronic (USA), Abbott/Saint Jude Medical (USA) or Boston Scientific (USA). Patients were followed from de novo device implantation onwards, and had bi-yearly follow-up visits. All patients <18 years old at device implantation were excluded. The requirement for written informed consent was waived by the Institutional Review Board. This study adheres to the reporting guidelines for Transparent Reporting of a multivariable prediction model for Individual Prognosis Or Diagnosis (TRIPOD), where applicable.^23^

### Outcome of interest

The outcome of interest was malignant ventricular arrhythmia, defined as an episode of sustained ventricular tachycardia or ventricular fibrillation, treated by the ICD through a shock and/or anti-tachycardia pacing (ATP). Outcomes were collected from the electronic health record (EHR).

### Clinical variables

Clinical baseline information at device implantation was extracted from the EHR, including medical history, medication usage (i.e. anti-arrhythmic, anti-coagulant, anti-hypertensive, lipid-lowering), demographics (age, sex), body mass index (BMI), and laboratory values (i.e. serum creatinine, potassium and sodium levels). Missing data were imputed using Random Forests for non-parametric imputation (missForest package (v. 1.1.3.) in Python).^24^ Variables with ≥30% missing values were excluded (Supplementary Table S1 shows missing values per variable). Categorical variables were one-hot encoded, and continuous variables were standardised using z-score.

### Electrocardiography

Raw-format, standard 12-lead 10-second resting ECGs were collected retrospectively on both sites. A total of 77,099 ECGs were retrieved, of which 23,423 from Hospital A and 53,676 from Hospital B. ECGs that were recorded at 500hz were downsampled to 250 Hz. Noise filtering and baseline wander removal was performed on the raw signals, by implementing a Savitzky–Golay Filter and subtracting low resolution Fourier series. In order to smooth data, the Savitzky–Golay filter deploys a low-pass filter that fits high-order polynomials at the local level through a least-squares technique.^25^ To eliminate baseline wander, a low-resolution Fourier cosine series reconstruction was employed by subtracting it from the original signal. Supplementary Figure S1 visually demonstrates the results of ECG filtering and noise reduction techniques. No visual inspection of ECGs was performed. These operations were performed using Python libraries NumPy (version 1.24.3), and tqdm (version 4.64.1). On both sites, the majority of ECGs have been recorded using GE Healthcare ECG devices (approximately 70% of ECGs). Other vendors were Welch Allyn, CSYS and DataM (all roughly 10%). In total, 44,629 ECGs (median 8 ECGs per patient [IQR 4–15]) were recorded during follow-up. Supplementary Figure S2a shows the distribution of the number of ECGs among patients. In cases where multiple ECGs were recorded on the same day, only the first ECG was selected, which resulted in 32,129 ECGs used within the dynamic framework. In total, 28% of ECGs were recorded within the first year of follow-up, 15% during the second year, 12% during third year, 10% during the fourth year, 8% during the fifth year (Supplementary Figure S2b). As wearable devices are currently only able to record the six (limb) leads of the 12-lead ECG (i.e. leads I, II, III, aVR, aVL and aVF), we used these in the present analysis.^26^ Individual heartbeats were isolated by automatic marking of individual R-peak locations, and subsequent extraction of heartbeat templates given a list of these locations. Mean waveforms were calculated by averaging individual waveforms per unique lead. ECG waveforms were pre-processed by normalising the signals between 0 and 1.

### Clinical ECG classifications

Human-interpretable, clinical ECG classifications were obtained using a deep neural network developed by Ribeiro et al., which provides an end-to-end learning approach to predict rhythm disorders and conduction disturbances from 12-lead raw ECG signals.^27^ A convolutional neural network (CNN) trained and tested on 2,322,513 ECGs from 1,676,384 patients was able to accurately predict six ECG abnormalities: 1st degree AV block, right bundle branch block (RBBB), left bundle branch block (LBBB), sinus bradycardia (SB), sinus tachycardia (ST) and atrial fibrillation (AF). We used the weights of this trained neural network to predict these classes for each of the 12-lead ECGs, which are publicly accessible. Alongside these classifications, standard ECG measurements, including ventricular heart rate, QRS-duration, QT-interval and PQ-time, were extracted.

### Variational autoencoder

We trained a custom **β-**VAE model (VAE for simplicity) to map information in the raw ECG waveforms into a lower-dimensional latent space, preserving important features. A plain autoencoder is a type of neural network commonly used in unsupervised learning that can learn a compact representation of high dimensional data. It consists of an encoder part, which maps the input into a latent space (normally of smaller dimension) designed to meaningfully represent the original data, and a decoder part, which takes that lower dimension latent space and aims to reconstruct the original input. A VAE adds a probabilistic component to the plain autoencoder network. Our VAE architecture was pre-trained on 256,205 ECGs (1,537,230 waveforms), to reach an initial state in the common domain of ECG signals morphologies. The pre-train ECG database consisted of routine ECGs collected at the general ward and outpatient clinic of the Amsterdam Medical Center between 1998 and 2018.^28^ The ECGs were extracted from the MUSEweb data management system (GE Healthcare, Chicago, Illinois, United States of America). The database includes one ECG per patient (mean age of 50 years, range 18–60 years, 52% male). Subsequently, this model was tuned on the ECGs of the ICD patient cohort.

In particular, the architecture of our VAE consists of a 3-layer encoder, with ReLu activation functions, ending into a mean and a standard deviation latent parameter layer, which parametrise a Gaussian distribution in the latent space. A Monte Carlo mechanism is used to provide a sample to feedforward the 3-layer symmetric decoder, which reconstructs the original ECG input. The loss function to train the VAE is comprised of two parts, as arises from the evidence lower bound (ELBO): the reconstruction error (for our data we used the mean square error, given the non-binary nature of the input) and the Kullback-Leibler (KL) divergence, both derived from the energy expression of the probabilistic model. The KL-divergence component of the loss function pushes the distribution of the encoder to be as close as possible to a multivariate normal distribution, regularising the latent space,^29^ providing our model with generalisability to other ECG recordings. The Monte Carlo sampling step ensures that the VAE learns a continuous and structured latent space.^29^^,^^30^ The decoder's task is to reconstruct the original input from these sampled latent representations. The combination of the reconstruction loss (how well the decoder reconstructs the input from the sampled latent representation) and the KL-divergence (aiming to regularise the latent space) ensures that the latent space captures the most salient and meaningful features of the input data.

Two VAEs were trained, one was trained on the waveforms from the six limb leads and the other was trained on lead II only, both encoding a latent space consisting of 16 variables. The performance of the VAE to reconstruct ECG waveforms was assessed by calculating the Pearson's correlation coefficient, root mean square Error (RMSE), percentage root mean square Distance (PRD) and dynamic time warping (DTW) between the original and the reconstructed signals. To match the studies by Beetz et al. (2022) and Zhu et al. (2019)^18^^,^^31^ we applied min–max normalisation to both the input and predicted ECG waveforms before calculating the metrics. VAEs were developed using Tensorflow Keras (version 2.13.1). The architectural details of the model, comprising a total of 343,692 parameters, are presented in Supplementary Figure S3.

### Time-varying cox regression

A Cox's time-varying proportional hazard model was used to examine associations between baseline clinical variables, time-varying latent ECG representations, and the outcome of interest.^32^ In order to incorporate time-varying covariates into the model, it was necessary to restructure the data into a person-period format, which enables the modification of feature values for a patient over time, by creating multiple rows where different feature values can be assigned.^33^ Hazard ratio's (HR) and confidence intervals (CI) were obtained.

### Random survival forests for dynamic and static models

Supervised ML survival models were trained, for which data was split in a training and a hold-out test set with an 80:20 ratio. Individual patients were assigned to either training or testing fold to prevent data leakage. The first model, a static random survival forest (RSF) model, incorporated clinical variables and ECG latent variables obtained at device implantation to estimate a personalised survival function. RSF is an ensemble of tree-based learners that predicts survival outcomes by aggregating the predictions of individual trees in the ensemble. We developed the RSF using the scikit-survival library (version 0.11).^34^ The second model, a dynamic model, integrated baseline data and time-varying ECG latent variables collected during follow-up. To develop the dynamic model, we utilised the Random Forest for Survival, Longitudinal, and Multivariate (RF-SLAM) methodology.^35^ RF-SLAM is a hybrid statistical and ML approach that uses a continuous-time random forest method for survival analysis to estimate individualised Bayes hazard rates. The input to the static RSF and dynamic RF-SLAM models was a combination of baseline clinical variables (medical history, medication usage, demographics, and laboratory values) and, respectively, baseline and dynamic ECG latent variables. Unlike the static RSF model, RF-SLAM requires longitudinal data to be binned in a pre-specified time interval (counting process information units, CPIU), allowing the predictor variables to change from one interval to another and the model to predict the probability of an event of interest during each CPIU. To apply this approach, the data were pre-processed by partitioning the data from record-per-subject into record-per-interval. The event time was defined as a discrete interval of 90 days during which the subject was continuously at risk of the event. These discrete intervals were set to align with the recommended timeframes outlined in the guidelines for remote patient management of individuals with an ICD.^36^ The use of CPIUs allows for the time-varying ECG features to change over time, whereas the baseline features remain static. A last value carried forward approach was used if no new ECG data was available for a discrete time period. Model probabilities derived for each of the CPIUs (individuals have different number of CPIUs depending on the follow-up duration) were used to obtain a piecewise-constant hazard function. A Poisson regression splitting criterion was used that does not require the proportional hazards assumption. If censoring or the outcome of interest occurred prior to the end of the 90 days period, the risk time was the time from start of the CPIU to the time of censoring or the outcome. We tuned the hyperparameters of the RF-SLAM and RSF models using a grid search, employing k-fold cross-validation (5 folds) on the development cohort. We tuned the number of trees, mtry (the number of variables randomly selected as candidates for splitting a node), and the terminal node size. Supplementary Table S2 provides the hyperparameters space and their corresponding final values. All analyses were performed using Python (version 3.6.7), except the RF-SLAM modelling was performed using R statistical software (version 3.6.2, R Core Team).^37^ The RF-SLAM methodology has been successfully applied during the COVID-19 pandemic to facilitate real-time prediction of clinical worsening by using longitudinal measurements of vital signs and laboratory values.^38^

### Sensitivity analyses

Sensitivity analyses was performed by training separate dynamic ML models on a combination of ECG human-interpretable classes and clinical variables and ii) latent variables obtained from a single lead ECG and clinical variables. Furthermore, two dynamic ML models were trained to predict i) all-cause mortality and ii) malignant ventricular arrhythmias treated by ICD shock (excluding arrhythmias treated by ATP).

### Latent space exploration and feature importance

**Fig. 1:**
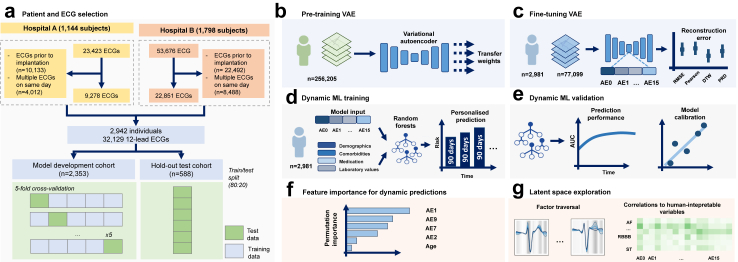
Schematic representation of data collection and model development. (a) Data collection and patient selection processes were independently carried out in two academic hospitals in The Netherlands. Electrocardiogram (ECG) retrieval was performed, and the dataset was subsequently divided into training and testing cohorts. (b) Pre-training of the Variational Autoencoder (VAE) using an extensive ECG database consisting of 256,205 recordings, optimised weights were then transferred to a subsequent VAE. (c) The second VAE underwent fine-tuning using a dataset comprising 77,099 ECGs, performance of the VAE in terms of reconstruction quality was evaluated using metrics such as root mean square error (RMSE), Pearson's correlation coefficient, dynamic time warping (DTW), and percentage root mean square difference (PRD) (d, e) A dynamic supervised machine learning model was trained and validated to predict malignant ventricular arrhythmias. This prediction was performed for each subsequent 90-day bin during a follow-up period spanning 48 months. (f) Feature permutation importance analysis was conducted to enhance the interpretability of the model's predictions. (g) The latent space of the model was explored, providing insights into the latent variables and contributing to the understanding of the model's behaviour and predictions.

Model explainability and interpretability are paramount for adoption in clinical practice. Within our proposed pipeline, there are two stages where model decisions are made through implicit learning: the unsupervised VAE for feature extraction from the ECG waveforms and the supervised dynamic ML for outcome prediction (Fig. 1f and g).

First, to better understand the low-dimensional representations that the VAE has mapped the ECG waveforms explored the latent space through a factor traversal. At the model level, we visualised the effect of individual latent space variables on the ECG morphology by varying the values of the individual latent space variables (5th, 25th, 75th and 95th percentiles of the original value), while reconstructing the signal using the decoder. Considering all other factors were kept constant during the procedure, we were able to visualise the effect each latent space variable potentially has on the morphology of a mean waveform. Additionally, to assess potential correlations between latent space variables and human-interpretable ECG measurements, we conducted an analysis to examine the relationships among latent space variables, the predicted probabilities associated with human-interpretable ECG classifications (such as 1st degree AV block, RBBB, LBBB, SB, ST, AF), and ECG measurements (such as ventricular heart rate, QRS duration, QT interval, and PQ time).

Second, we assessed permutation feature importance to better explain predictions of the dynamic ML model. Permutation feature importance measures the impact of permuting the values of individual features to identify which contribute most to model performance. We repeated permutations five times per feature (both latent space variables and clinical features), and calculated the average feature importance. A higher positive score suggests that the feature is important for model predictions, while a lower or negative score indicates the converse.

### Statistics

Continuous variables were presented by its median, mean, interquartile range (IQR) and standard deviation (SD). Categorical sociodemographic and clinical variables were presented as frequencies (percentages) and compared using Fisher's exact test when appropriate, otherwise using Chi-square test. For each individual time point, the time-specific receiver operating characteristics (ROC) curve and area under the ROC curve (AUROC) were obtained. From this, the time-dependent AUROC was created, defined as the area under the time-specific ROC curve. Model calibration was evaluated with the decile method, in which predicted risks are grouped into 10 deciles. Performance was assessed on the hold-out test set after model optimisation on the training set, evaluated on 500 iterations of bootstrapped test sets where unique patient samples were randomly drawn with replacement. Probabilistic models, although effective in making inferences based on patterns within large datasets, are susceptible to systematic unfairness, which can result in disparate predictions across different subgroups. To evaluate the fairness of predictions, we compared model performance within the following subpopulations: male versus female patients, ischaemic cardiomyopathy versus non-ischaemic cardiomyopathy, primary versus secondary prevention ICD indication, and patients with a LVEF below 35% versus LVEF above 35%.

### Ethics

The requirement for written informed consent for this retrospective study was waived by the Institutional Review Board, as the medical research involving Human Subjects Act did not apply.

### Role of the funding source

The funding source had no role in the study design, data collection, data analyses, interpretation, or writing of report.

## Results

A total of 2942 patients were included in the analysis, with 1144 from Hospital A and 1798 from Hospital B (Fig. 1). As summarised in Table 1, 1225 (41.6%) had an ischaemic cardiomyopathy, 678 (23.0%) a dilated cardiomyopathy and 186 (6.3%) a hypertrophic cardiomyopathy. A total of 905 (30.8%) patients were diagnosed with an atrial arrhythmia, 578 (19.6%) with diabetes mellitus and 360 (12.2%) had suffered a cerebral vascular accident. The majority of patients (76.6%) used a β-blocker at baseline. Patients received one of four types of ICDs: single-chamber (36.6%), dual-chamber (26.8%), subcutaneous ICD (13.6%), or CRT-D (23.1%). During a mean follow-up of 43.9 months ± 35.9, 840 (28.6%) patients had a malignant ventricular arrhythmia treated by the ICD: 15.0% received an appropriate shock, 7.2% ATP followed by shock and 6.4% only ATP (Table 2). A total of 631 (21.4%) patients died during the follow-up period. Supplementary Figure S4 shows the survival curves for the outcome of interest, and all-cause mortality. The average Pearson correlation coefficient between the original and the reconstructed ECG waveforms was 0.93 ± 0.09, the RMSE was 0.0495 ± 0.026, the PRD was 9.49 ± 5.03 and DTW was 4.61 ± 2.90. An example of a reconstructed signal is depicted in Supplementary Figure S5. The single-lead VAE reconstructed signals with a Pearson's correlation coefficient of 0.99 ± 0.01.

**Table 1:** Baseline characteristics.

	Total cohort (n = 2942)	Hospital A (n = 1144)	Hospital B (n = 1798)	*p* value
Age, mean (SD)	61.7 (13.9)	64.3 (12.0)	60.1 (14.8)	<0.001
Male, yes (%)	2193 (74.5)	878 (76.7)	1315 (73.1)	0.032
**Primary prevention ICD indication, yes (%)**	1779 (60.5)	712 (62.2)	1067 (59.3)	0.127
*LVEF, yes (%)*				
>45%	671 (22.8)	146 (12.8)	525 (29.2)	<0.001
35–45%	489 (16.6)	250 (21.9)	239 (13.3)	
≤35%	1782 (60.6)	748 (65.4)	1034 (57.5)	
OHCA, yes (%)	814 (27.7)	336 (29.4)	478 (26.6)	0.109
**Prior ventricular arrhythmia, yes (%)**				
None	1410 (47.9)	585 (51.1)	825 (45.9)	<0.001
Ventricular fibrillation	792 (26.9)	331 (28.9)	461 (25.6)	
Sustained VT	403 (13.7)	123 (10.8)	280 (15.6)	
Non-sustained VT	337 (11.5)	105 (9.2)	232 (12.9)	
**Cardiomyopathy, yes (%)**				
Ischaemic	1225 (41.6)	608 (53.1)	617 (34.3)	<0.001
Dilated	678 (23.0)	326 (28.5)	352 (19.6)	
Hypertrophic	186 (6.3)	46 (4.0)	140 (7.8)	
Non-ischaemic	158 (5.4)	12 (1.0)	146 (8.1)	
Primary arrhythmia syndrome	68 (2.3)	26 (2.3)	42 (2.3)	
**Medical history, yes (%)**				
Percutaneous coronary intervention	991 (33.7)	442 (38.6)	549 (30.5)	<0.001
Coronary artery bypass grafting	456 (15.5)	203 (17.7)	253 (14.1)	0.008
Myocardial infarction	1363 (46.3)	572 (50.0)	791 (44.0)	0.002
Atrial arrhythmia	905 (30.8)	337 (29.5)	568 (31.6)	0.238
CVA	360 (12.2)	135 (11.8)	225 (12.5)	0.605
COPD	212 (7.2)	91 (8.0)	121 (6.7)	0.238
Diabetes mellitus	578 (19.6)	251 (21.9)	327 (18.2)	0.014
BMI, mean (SD)	27.1 (4.2)	27.1 (4.6)	27.1 (4.0)	0.639
Peripheral artery disease	164 (5.6)	67 (5.9)	97 (5.4)	0.653
Hypertension	1262 (42.9)	579 (50.6)	683 (38.0)	<0.001
CHD	80 (2.7)	10 (0.9)	70 (3.9)	<0.001
**Laboratory, mean (SD)**				
Sodium, mmol/L	139.5 (2.9)	139.5 (3.0)	139.5 (2.7)	0.885
Potassium, mmol/L	4.3 (1.0)	4.3 (0.5)	4.2 (1.2)	0.155
Creatinine, umol/L	100.2 (60.7)	101.2 (58.4)	99.6 (62.2)	0.501
**Medication, yes (%)**				
Vitamin K antagonist	781 (26.5)	398 (34.8)	383 (21.3)	<0.001
NOAC	286 (9.7)	79 (6.9)	207 (11.5)	<0.001
Sotalol	173 (5.9)	35 (3.1)	138 (7.7)	<0.001
Digoxin	171 (5.8)	72 (6.3)	99 (5.5)	0.418
Amiodarone	258 (8.8)	92 (8.0)	166 (9.2)	0.296
β-blocker	2253 (76.6)	959 (83.8)	1294 (72.0))	<0.001
Mineralocorticoid receptor antagonist	783 (26.6)	382 (33.4)	401 (22.3)	<0.001
Diuretic	1351 (45.9)	51.2 (58.6)	765 (42.5)	<0.001
ARB/ACEi,	2089 (71.0)	904 (79.0)	1185 (65.9)	<0.001
GDMT^a^	542 (18.4)	281 (24.6)	261 (14.5)	<0.001
**Device, yes (%)**				<0.001
Single-chamber ICD	1076 (36.6)	373 (32.6)	703 (39.1)	
Dual-chamber ICD	788 (26.8)	411 (35.9)	377 (21.0)	
CRT-D	679 (23.1)	297 (26.0)	382 (21.2)	
S-ICD	399 (13.6)	63 (5.5)	336 (18.7)	

Abbreviations: ARB: Angiotensin receptor blockers, ACEi: angiotensin-converting-enzyme inhibitors, BMI: body mass index, CHD: congenital heart disease, COPD: chronic obstructive pulmonary disease, CRT: cardiac resynchronisation therapy, CVA: cerebral vascular accident, GDMT: guideline-directed medical therapy, ICD: implantable cardioverter-defibrillator, LVEF: left ventricular ejection fraction, NOAC: novel oral anticoagulants, OHCA: out-of-hospital cardiac arrest, SD: standard deviation, S-ICD: subcutaneous ICD, VT: ventricular tachycardia.

a β-blocker, angiotensin-converting enzyme (ACE) inhibitors/angiotensin receptor blockers inhibitors, and a mineralocorticoid receptor antagonists (MRA).

**Table 2:** Event rates during follow-up.

	Total cohort (n = 2942)	Hospital A (n = 1144)	Hospital B (n = 1798)	*p* value
**Malignant ventricular arrhythmia, yes (%)**	840 (28.6)	314 (27.4)	526 (29.3)	0.310
Treated by shock	441 (15.0)	165 (14.4)	276 (15.4)	
Treated by ATP	187 (6.4)	88 (7.7)	99 (5.5)	
Treated by shock and ATP	212 (7.2)	61 (5.3)	151 (8.4)	
**All-cause mortality, yes (%)**	631 (21.4)	276 (24.1)	355 (19.7)	0.005

Abbreviations: ATP: anti-tachycardia pacing.

### Cox's time varying proportional hazard model

In multivariable time-varying Cox regression, latent space variables *AE8* and *AE0* were significantly associated with the outcome of interest (HR 1.19, 95% CI 1.01–1.40, *p* = 0.036, HR 1.19, 95% CI 1.01–1.41, *p* = 0.034, respectively). In addition, significant associations were observed for a prior sustained VT or VF, dilated and ischaemic cardiomyopathy, male sex, reduced LVEF and sotalol usage. Cardiac resynchronisation therapy was found to have a negative association with malignant ventricular arrhythmias, with a HR of 0.72 (95% CI 0.59–0.89, *p* < 0.001). Sensitivity analyses demonstrated no significant associations between the outcome of interest and latent space variables derived from single-lead ECG or the six human-interpretable ECG classifications.

### Predictive performance of dynamic and static models

**Fig. 2:**
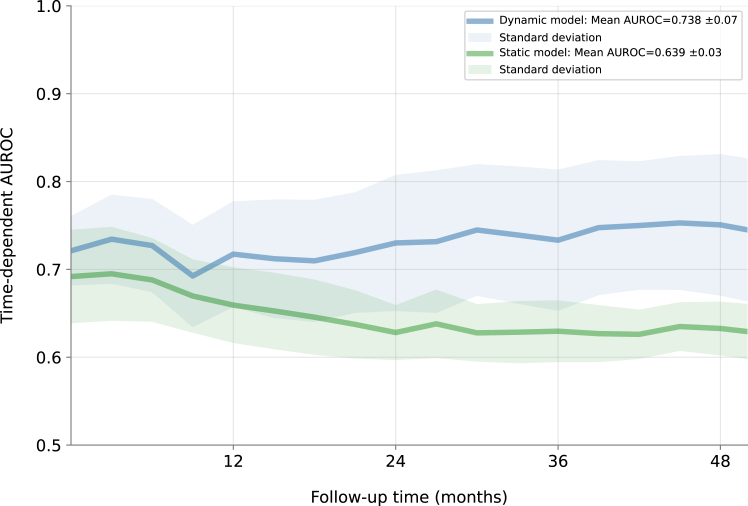
Time-varying area under the ROC curve for the dynamic and static machine learning models in the hold-out test set. The discriminative ability of the dynamic model trained on time-varying ECG data increased over time, while that of the static model trained on baseline information only falls. *p*-values indicating statistical differences in time-varying area under the ROC were consistently below 0.05, starting from the 12-month follow-up onwards.

The time-dependent AUROC for the dynamic model and static model are visualised in Fig. 2. For the dynamic model, the mean time-dependent AUROC on the hold-out set was 0.738 ± 0.07, compared to 0.639 ± 0.03 for the static model. Visual inspection of the AUROC over time demonstrated that the discriminative ability of the dynamic model increased over 2–4 years of follow-up, while that of the static model fell. Model calibration assessed by risk decile showed good agreement between the predicted probabilities and the observed outcomes (Supplementary Figure S6). Sensitivity analyses indicated dynamic models trained on latent space variables from single-lead ECGs and human-interpretable ECG classifications had lower AUROCs, respectively 0.688 ± 0.07 and AUROC 0.694 ± 0.08 (Supplementary Figures S7 and S8). The dynamic ML model predicted all-cause mortality with a mean time-varying AUROC of 0.782 ± 0.07 (Supplementary Figure S9), and malignant ventricular arrhythmias treated by ICD shock only with a mean time-varying AUROC of 0.743 ± 0.07 (Supplementary Figure S10). Model performance in subpopulations showed higher AUROC in female patients compared to male patients (Supplementary Figure S11), while performance was similar for other subgroups.

### Feature importance and latent space exploration

**Fig. 3:**
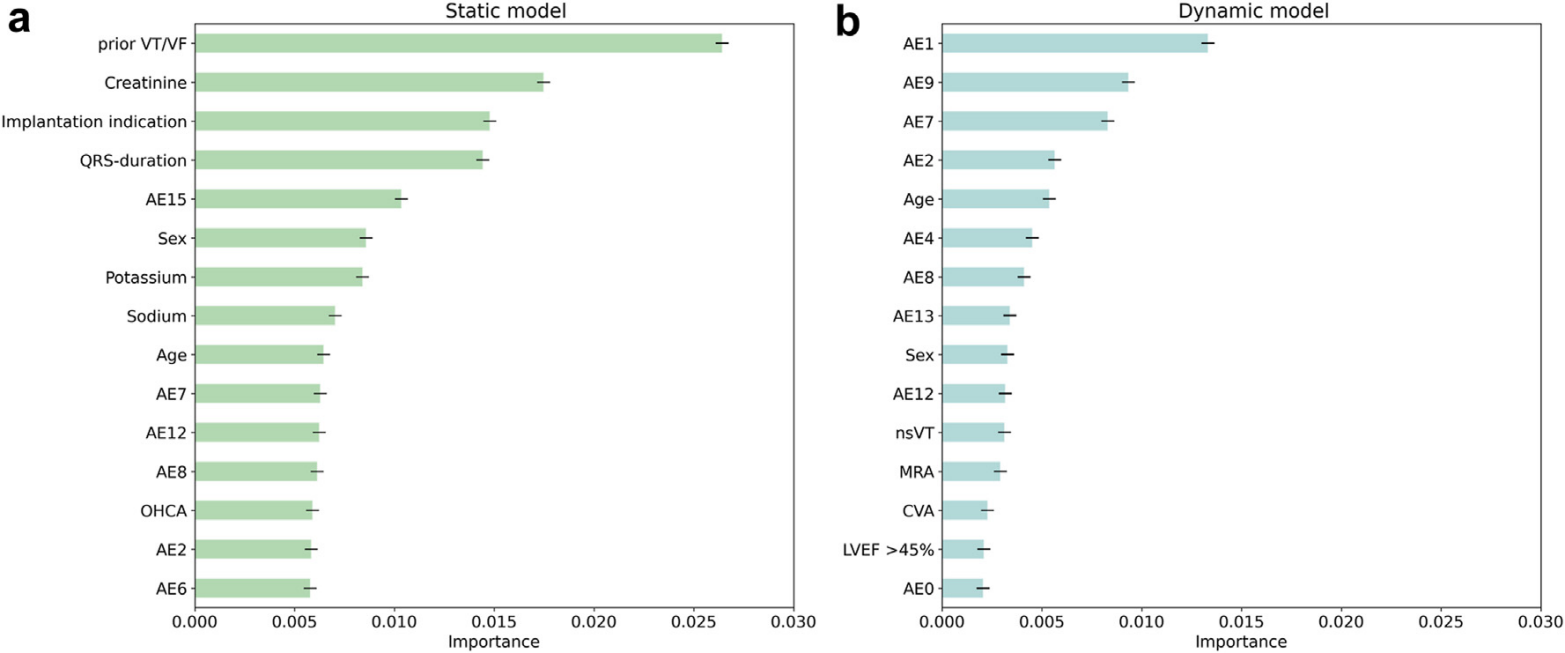
Feature permutation importance for the static (a) and the dynamic (b) machine learning models for the prediction of malignant ventricular arrhythmias. Permutation feature importance gauges the perturbation impact of individual feature values on the model's predictive error, to identify features that affect the model's performance the most. The permutation procedure was iterated five times for each feature category (latent space variables and clinical attributes). A higher positive score denotes greater importance in the model's predictions, whereas a lower or negative score indicates potential detriment to the model's predictive performance. Abbreviations: AE: autoencoder, BMI: body mass index, LVEF: left ventricular ejection fraction, MRA: mineralocorticoid receptor antagonist, OHCA: out-of-hospital cardiac arrest, Prior VT/VF: any prior episode of ventricular tachycardia or fibrillation prior to ICD implantation, implantation indication: reason for ICD implantation (primary prevention versus secondary of sudden cardiac death). Potassium, sodium and creatinine were numeric serum levels.

**Fig. 4:**
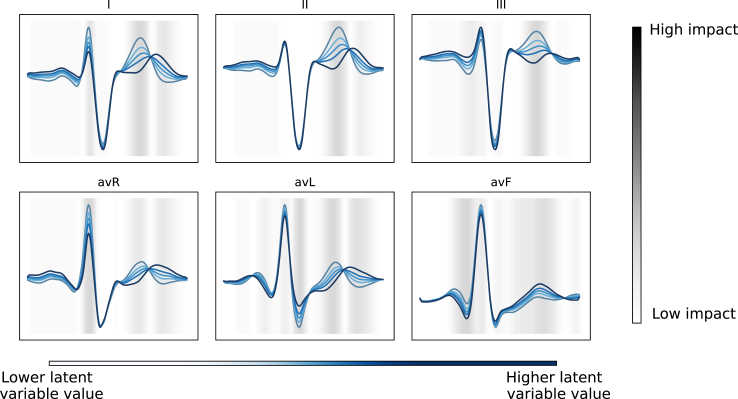
Visualisation of the morphological changes in the electrocardiographic waveform by factor traversal analysis of the latent space variable (*AE1*). Latent space variables were changed to the 5th, 25th, 75th and 95th percentile of the original value. The altered latent space vectors were passed through the decoder to generate reconstructed waveforms. The heatmap represents the magnitude of the differences in the ECG morphologies (i.e. the regions of the ECG mean waveform with greatest variation) between latent variables.

In Fig. 3, feature permutation importance is presented for both the static model and the dynamic model. With respect to the dynamic model (Fig. 3, panel b), high permutation importance was observed for latent space variables (*AE1*, *AE9*, *AE7*, *AE2*) and age. In the static model (Fig. 3, panel a), features that demonstrated highest permutation importance were prior malignant ventricular arrhythmia(s), implantation indication and serum creatinine levels. Fig. 4 shows the morphological changes in the ECG waveform during traversal of the latent space, in which values of latent space variable *AE1* were systematically changed while keeping other variables constant, and the altered latent space was subsequently passed through the decoder to generate reconstructed waveforms. Factor traversal of *AE1* affected in particular the morphology of the T-wave, reflecting ventricular repolarisation. Supplementary Figure S12 displays the latent space traversal for each latent space variable. Correlations between human-interpretable ECG classifications and measurements, and latent space variables, are depicted in Supplementary Figure S13. Moderate correlations were observed for RBBB, LBBB, sinus bradycardia and sinus tachycardia, while atrial fibrillation, 1st degree AV block and ECG measurements exhibited weak correlations with latent space variables. Latent space variables with moderate correlations to clinical ECG classifications (*AE0*, *AE3*, *AE8* and *AE10*), displayed low feature importance for dynamic predictions.

## Discussion

We present the training and validation of a dynamic ML model that integrated baseline covariates and time-varying deep learning latent ECG representations for the prediction of malignant ventricular arrhythmias. Using a variational autoencoder, low-dimensional latent representations were extracted from the ECG, which potentially reflect ventricular arrhythmic risk and may not be captured by standard clinical ECG interpretations. Unlike static models, this dynamic model was capable of providing updated predictions over time, capturing disease trajectories and handling complex non-linear relationships among predictors. Overall, our findings suggest that integrating the dynamics between triggering mechanisms and static ventricular arrhythmic substrates captured in the ECG can be represented by deep learning models to accurately predict onset of malignant ventricular arrhythmias.

The exponential increase in personalised and continuously collected data through digital health tools (e.g. wearable sensors, remote device monitoring), has led to a growing interest in models designed to update predictions over time.^10^^,^^39^, ^40^, ^41^ Highly granular physiological data can be leveraged by ML models to detect intricate and non-linear interactions, revealing patterns and associations that may not be apparent through traditional statistical methods.^8^ In the context of cardiac arrhythmogenesis, where structural cardiac abnormalities provide the substrate on which transient risk factors operate, dynamic models using data collected real-time or at frequent intervals are particularly pertinent.^6^^,^^7^^,^^42^ Machine learning (e.g. RF-SLAM, LTRC forests) and deep learning (e.g. DeepHit, DeepSurv) methodologies that facilitate the integration of time-varying covariates for outcome prediction have become more widely available over the past decade.^43^, ^44^, ^45^ Among the first studies to address the potential advantage of time-varying covariates and updated predictions in terms of the predictive accuracy was by Wu et al., who validated a ML model that incorporated serial measurements from cardiac magnetic resonance imaging (CMR) in 382 primary prevention ICD patients. This dynamic model yielded a mean time-dependent AUROC of 0.88 (95% CI 0.75–0.96), outperforming static models during internal validation (no independent validation of this model was performed).^46^ As opposed to CMR, ECG serves as a widely available, cost-effective and non-invasive alternative to cardiac imaging techniques which may even be acquired in remote settings through the use of wearable devices. Deep learning approaches, in particular, have potential to extract and process features from high dimensional, complex electrophysiological signals unrecognisable to human-eye, and use these to predict arrhythmia risk.^8^^,^^47^ Kwon et al. trained and validated a convolutional neural network for predicting in-hospital cardiac arrest on 32,294 ECGs, which yielded an AUROC of 0.930 on an external, retrospectively collected patient cohort.^11^ However, despite high discriminative power, the positive predictive value at the point of high sensitivity was only 8%. In their study, Perez-Alday et al. investigated the potential of longitudinal, hand-crafted ECG metrics for predicting arrhythmia onset, and observed that the prognostic accuracy of these metrics, particularly for events happening within 3–9 months, was low.^48^ We hypothesised that by employing a ML approach that integrated low-dimensional latent ECG representations extracted from longitudinal ECGs, we would be able to better capture arrhythmic substrates and triggers and use these for accurate arrhythmia prediction.^19^^,^^49^ The improved predictive performance of the dynamic model over static models, and these predictions being predominantly driven by time-varying latent ECG representations, points towards the potential benefits of integrating temporal variability of covariates within prediction models. Moreover, prior studies have demonstrated deep learning models specifically designed to handle sequential data, for example recurrent neural networks, are able to capture the complex patterns within large longitudinal datasets.^40^ This differs from our approach, where we demonstrated that we can effectively extract the pertinent features that summarise the ECG waveforms using neural networks, and use these in a simpler supervised ML model to predict outcomes. This is akin to using a pure deep learning model, but with more controlled features in a well-regulated environment, which may be preferable in situations where the number of cases is low. In addition, while supervised deep learning models excel at capturing complex patterns and intricate relationships in the data these models are faced with the critical trade-off between explainability and performance, in contrast ensemble methods tend to be more interpretable. Using a two-staged methodology which harnesses the ability of deep learning for feature extraction and representation learning, while also benefiting from the transparency and interpretability of traditional machine learning models, we were able to gain a deeper understanding of the underlying relationships between time-varying ECG data and baseline clinical information.

By utilising the strength of neural networks and probabilistic models, variational autoencoders are capable of learning to compress any given ECG signal into a set of explanatory and independent factors, allowing them to operate as generative networks. Our model provided reconstructed original ECG signals favourably compared to the literature (Supplementary Table S3). Various convolutional models have been developed to represent ECG waveforms,^18^^,^^20^^,^^49^ and 10-s ECG signals.^19^ Conversely, our relatively simple VAE with only 3 fully connected layers reconstructed mean 6-lead waveforms with a Pearson's correlation coefficient of 0.93 ± 0.09. We accept that while these results compare favourably with previously published models, there were substantial differences in datasets and in pre-processing steps (Supplementary Table S3) and should be regarded the basis for future comparisons.

The predictive capacity of latent space variables for arrhythmia prediction reportedly outperforms standard ECG criteria and clinical interpretations, which indicates the ability of autoencoders to map unique prognostic ECG features to a latent representation not reflected by traditional ECG parameters.^17^^,^^18^^,^^20^^,^^21^ The framework used in this study, which integrates an unsupervised learning branch (the autoencoder) and a supervised learning branch (the dynamic ML model), is related to the concept of *digital twins*, which create virtual replicas of individual patients by continuously integrating and analysing their real-time physiological data.^50^^,^^51^ Despite the potential of deep learning networks to extract features from high dimensional data and aid clinical decision-making, the interpretability and explainability of neural networks remains a significant challenge and an active field of research. While latent space variables can reflect clinically-relevant morphological changes in the ECG, such as ST depression and T-wave amplitude, not every feature in the latent space has a direct one-to-one correspondence with a specific clinical change.^19^^,^^20^^,^^49^ To better understand the nature of the latent space variables, we used a trained CNN to obtain human-interpretable ECG classifications alongside traditional ECG measurements and correlated them with the latent features. We found only moderate correlations between latent space variables and human-interpretable ECG classifications, in particular the presence of bundle branch blocks (LBBB and RBBB) and an abnormal heart rate (sinus bradycardia and tachycardia). Second, we employed a factor traversal technique to explore the latent space and visualise the contribution of each variable to the (reconstructed) ECG morphology. Notably, we observed that latent space variables ranked highest in terms of feature importance (*AE0, AE7*, *AE8*, *AE9*) affected the morphology of the T-wave, in particular inversion of the T-wave in lead III. Abnormalities in ventricular repolarisation, represented by the T-wave, have been associated with ventricular arrhythmic substrates such as myocardial fibrosis, and may arguably explain the relevance of these particular latent space variable for the dynamic model.^52^, ^53^, ^54^, ^55^ Related to this is the notion that there may be cardiac pathologies where beat-to-beat variations changes in the T-wave morphology are observed (e.g. due to ventricular ectopy), although it is not clear whether these variations are captured during the encoding process. Moreover, feature permutation importance analyses of the static and dynamic ML models showed some notable differences. Although both models incorporated the same clinical variables, the dynamic ML model predictions were mainly driven by time-varying latent variables extracted from the ECG, while the static model built at a single snapshot in time validated well-known features of other models including prior VT or VF, indication for ICD implantation, elevated serum creatinine, prolonged QRS-duration, age and sex. These findings underscore the exciting potential to improve upon malignant ventricular arrhythmia prediction using continuous data streams such as are increasingly available from consumer wearables as well as clinical monitors. Future studies may assess the impact of incorporating both time-varying ECG information and changes in clinical variables over time on the performance of such models. All in all, the superior accuracy of the model trained using latent space variables, compared to human-interpretable ECG classifications, suggests that autoencoders capture prognostically relevant ECG features, not reflected by traditional human-interpretable ECG interpretations.

### Limitations

There are several limitations to acknowledge. First, due to the retrospective nature of this study, details on the programming of the ICD (i.e. detection times and zones) were not available, although variations in device programming may affect the incidence of ICD-therapy for malignant ventricular arrhythmias. Second, prior studies have identified predictors for ventricular arrhythmias from imaging modalities, including CMR and echocardiography.^46^^,^^56^ From this study we are not able to fully explain the extent to which the autoencoder has captured features from ECG that reflect information from these domains, such as left ventricular functionality and myocardial fibrosis. Of note, autoencoders have been applied to learn latent representation of 3D cardiac anatomy that, potentially in combination with ECG, could be used to train multi-domain autoencoders with a shared latent space.^18^^,^^57^^,^^58^ Third, we have trained our models on a selected population of ICD carriers, with a high a priori probability of malignant ventricular arrhythmias, which affects the generalisability of the model to other populations. We explored the risk of unrecognised bias leading to ‘unfair’ predictions by evaluating model performance within clinically-relevant subgroups, however, it is imperative that future studies assess the robustness of this model prospectively, and across external patient cohorts, taking into account potential biased behaviours in underrepresented groups.^59^ We were able to include data from two different settings in the current study, however, model validation on external patient cohorts are crucial to assess the robustness of the model. Fourth, latent traversal, employed to evaluate the impact of individual latent variables on the ECG morphology, depicts their effects on a standardised waveform and does not provide explainability on a patient-level. Factor traversal in autoencoders assumes that the latent factors are independent attributes or features, although autoencoders may exhibit interactions between latent factors. It remains to be studied if such limitations, are still more explainable than deep learning the massively parallel connections with non-linear relationships of deep neural networks. Future work should map latent factors to inputs for clinical interpretability, since it is difficult to relate factor traversal to specific semantic attributes. These factors contribute to an incomplete disentanglement and a semantic gap, limiting the interpretability of factor traversal in autoencoders. Last, ML techniques fail to provide insights in underlying mechanisms as these seek correlation rather than causality, which compromises the interpretability of model predictions. The integration of ML to find correlations in very large datasets and multiscale modelling to identify causality and mechanisms could proposedly be the next step towards explainable dynamic predictions.^60^

### Conclusion

Utilising dynamic ML models and variational autoencoders, prognostic information from routinely collected ECGs can be extracted and leveraged to provide personalised predictions of malignant ventricular arrhythmias in patients with an ICD. This approach has potential for integration within settings where ECG data is collected real-time or at frequent intervals, for instance through wearable sensors or implanted cardiac devices. Before clinical adoption, future studies are warranted to prospectively and externally validate these findings.

## Contributors

MK, SR, RK, SN and FT contributed to the conception and design of the study. MK, SR, BD, PC, SN and FT collectively investigated the data and decided on the methodology to be used. MK and SR conducted the formal analyses. MK, SR, RK, SN and FT drafted the original manuscript. MK, SR, LAF, BD, EB, CA, AL, II, PC, AW, RK, SN and FT reviewed, edited, and agreed with the final version of the manuscript. MK, SR and FT accessed and verified the underlying data.

## Data sharing statement

Data sharing requests will be considered upon a reasonable request. For access, please email the corresponding author. Code scripts for ECG pre-processing, time-series feature extraction and VAE model are available at: https://github.com/DeepRiskAUMC/Time-varying-predictions-of-VA.

## Declaration of interests

S.M.N. reports grant or contracts from National Institutes of Health (NIH), consulting fees from Abbott Inc. and royalties and licenses from Uptodate. R.E.K. reports consultancy fees and research grants from Boston Scientific, Medtronic and Abbott Inc. and has stock options from AtaCor Medical Inc. F.V.Y.T. has grants or contracts from the Dutch Research Council (NWO) and Amsterdam Cardiovascular Sciences, and received honoraria fees from Boston Scientific and Abbott Inc. (no personal financial gain). A.A.W. has consultancy fees from ARMGO and Thyrv Therapeutics, and participates on a Data Safety Monitoring Board or Advisory Board for the LEAP trial. C.P.A. reports grants or contracts from Biotronik. I.I. reports research grants or contracts from participation of Philips, Quantib, Pie Medical Imaging and Esaote, has patents planned, issued or pending, has stock option from RadNet and has leadership or fiduciary for Chair Medical Imaging with Deep Learning (MIDL) board, MIDL Foundation and SPIE Medical Imaging, Image processing. M.Z.H.K., S.R.C., B.D., L.A.F., E.J.B., P.C., and A.C.J.L have nothing to declare.
