# Supplementary material for: Dynamic prediction of malignant ventricular arrhythmias using neural networks in patients with an implantable cardioverter-defibrillator
Source: eBioMedicine. 2023 Dec 19;99:104937. doi: 10.1016/j.ebiom.2023.104937 (PMC10772563; doi:10.1016/j.ebiom.2023.104937)
Supplement: Supplementary Tables and Figures [file mmc1.docx]

**SUPPLEMENTARY MATERIAL**

Supplementary Tables

Supplementary Table 1. Missing values for the clinical variables

Supplementary Table 2. Hyperparameters search space for the RF-SLAM and Random Survival Forest models

Supplementary Table 3. Reconstruction performance of the variational autoencoder compared to previously published models for ECG reconstruction

Supplementary Figures

Supplementary Figure 1. Visualisation of signal pre-processing applied to the electrocardiograms (ECG)

Supplementary Figure 2. Distribution of the number of ECGs during follow-up per individual patient (A) and the duration between ICD implantation and the ECG recording (B)

Supplementary Figure 3. Schematic overview of variational autoencoder (VAE) architecture components

Supplementary Figure 4. Survival curves for the outcomes of interest

Supplementary Figure 5. Example of the original 6-lead ECG (blue) and a reconstructed signal (orange) using the variational autoencoder (VAE)

Supplementary Figure 6. Calibration plot of the dynamic model

Supplementary Figure 7. Time-dependent area under the curve (AUROC) for the 6-lead and the single-lead dynamic models

Supplementary Figure 8. Time-dependent area under the curve (AUROC) for the dynamic models trained on VAE-derived latent space variables or clinical ECG interpretations

Supplementary Figure 9. Time-dependent area under the curve (AUROC) for the prediction of all-cause mortality compared to malignant ventricular arrhythmias

Supplementary Figure 10. Time-dependent area under the curve (AUROC) for the prediction malignant ventricular arrhythmias treated by shock and/or anti-tachycardia pacing (ATP) versus shock-only

Supplementary Figure 11. Validation of model performance in patient subgroups

Supplementary Figure 12. Factor traverse for each latent space variable

Supplementary Figure 13. Heatmap showing the correlations between the latent space variables and the human-interpretable ECG interpretations and measurements

**Supplementary Table 1.** Missing values for the clinical variables

| **Variable** | **Count** | **Percentage** |
| --- | --- | --- |
| Age | 0 | 0% |
| Sex | 0 | 0% |
| Atrial arrhythmia | 0 | 0% |
| Left ventricular ejection fraction (LVEF) | 0 | 0% |
| OHCA | 0 | 0% |
| Cardiomyopathy | 0 | 0% |
| PCI | 0 | 0% |
| CABG | 0 | 0% |
| Myocardial infarction | 0 | 0% |
| VF | 0 | 0% |
| CVA | 0 | 0% |
| COPD | 0 | 0% |
| Diabetes Mellitus | 0 | 0% |
| BMI | 640 | 21.8% |
| PAF | 0 | 0% |
| QRS-Duration | 114 | 3.9% |
| Hypertension | 0 | 0% |
| CHD | 0 | 0% |
| Sodium | 590 | 20.1% |
| Potassium | 568 | 19.3% |
| Creatinine | 421 | 14.3% |
| Device type | 0 | 0% |
| ARB | 424 | 14.4% |
| Diuretic | 427 | 14.5% |
| Vitamin K antagonist | 431 | 14.7% |
| Sotalol | 434 | 14.8% |
| Digoxin | 434 | 14.8% |
| Amiodarone | 433 | 14.7% |
| Beta-blocker | 423 | 14.4% |
| NOAC | 435 | 14.8% |
| Aldosterone inhibitor | 433 | 14.7% |

**Supplementary Table 2.** Hyperparameters search space for the RF-SLAM and Random Survival Forest models

| **Model** | **Hyperparameters** | **Tuning values** | **Final parameter value** |
| --- | --- | --- | --- |
| **RF-SLAM** | Number of trees | [50, 100, 150, 200] | 100 |
|  | Mtry | [2, 4, 6] | 2 |
|  | Node size | [1.5, 2.5, 3.0] | 2.5 |
|  | Split rule | *Poisson split 1*: with stratification by risk time and interval time  *Poisson split 2*: with stratification by risk time but not interval time  *Poisson split 3*: with stratification by interval time but not risk time. | Poisson split 1 |
| **Random survival forest** | Number of estimators | [50, 100] | 100 |
|  | Maximum depth | [5, 10, 20, None] | 20 |
|  | Minimum number of samples at internal node | [2, 5, 10] | 5 |
|  | Minimum number of samples at leaf node | [1, 2, 4] | 2 |
|  |  |  |  |

**Supplementary Table 3.** Reconstruction performance of the variational autoencoder compared to previously published models for ECG reconstruction

|  | **ECGs** | **Model architecture** | **Root Mean Squared Error** | **Percentage Root Mean Squared Distance** | **Pearson correlation coefficient** | **Dynamic Time Warping** |
| --- | --- | --- | --- | --- | --- | --- |
| Beetz et al. (2022) ^1^ | 1300 median beats lead II | 2D CNN-VAE | 0.16 | 26.51 | N/A | N/A |
| Zhu et al. (2019) ^2^ | 48 single lead | BiLSTM-CNN GAN | 0.28 | 66.41 | N/A | N/A |
| Van der Leur et al. (2022) ^3^ | 1,144,331 median beats 12-lead | β-VAE | N/A | N/A | 0.90 | N/A |
| Delaney et al. (2019) ^4^ | 46 single lead 10-second ECG | 1CNN BiLSTM GAN | N/A | N/A | N/A | 7.296 |
| Current study | Pre-train 256,205 / Fine-tuning cohort 77,099 mean 6-lead ECG waveforms | β-VAE | 0.050 ±0.026 | 9.49 ±5.03 | 0.93 ±0.09 | 4.61 ±2.90 |
| *Abbreviations*: CNN= convolutional neural network, ECG=electrocardiogram, GAN=generative adversarial network, LTSM = long short-term memory, VAE=variational autoencoder | | | | | | |
|  |  |  |  |  |  |  |

**Supplementary Figure 1. Visualisation of signal pre-processing applied to the electrocardiograms (ECG)**

**
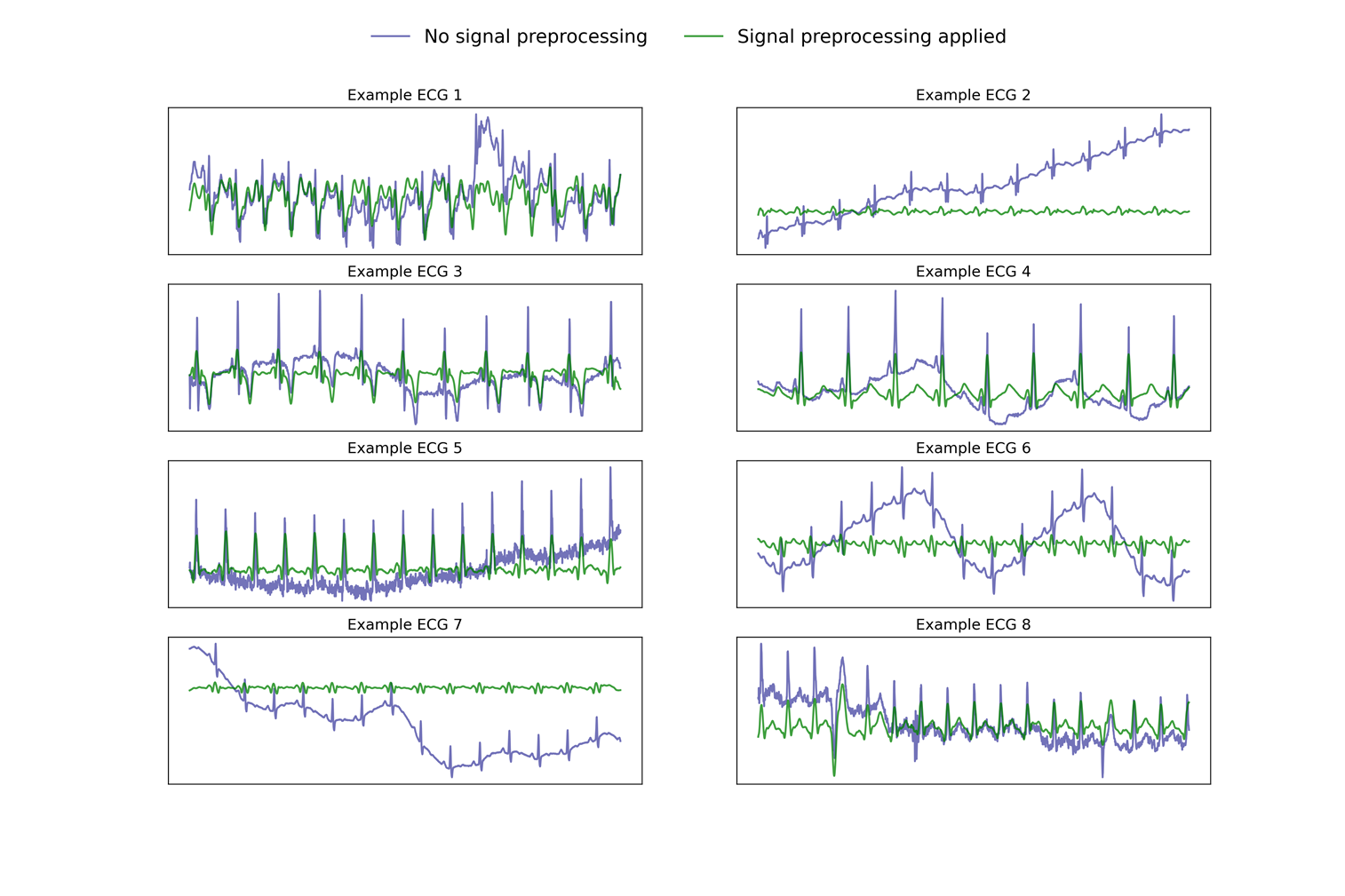
**

**Supplementary Figure 2**. **Distribution of the number of ECGs during follow-up per individual patient (a) and the duration between ICD implantation and the ECG recording (b)**

**
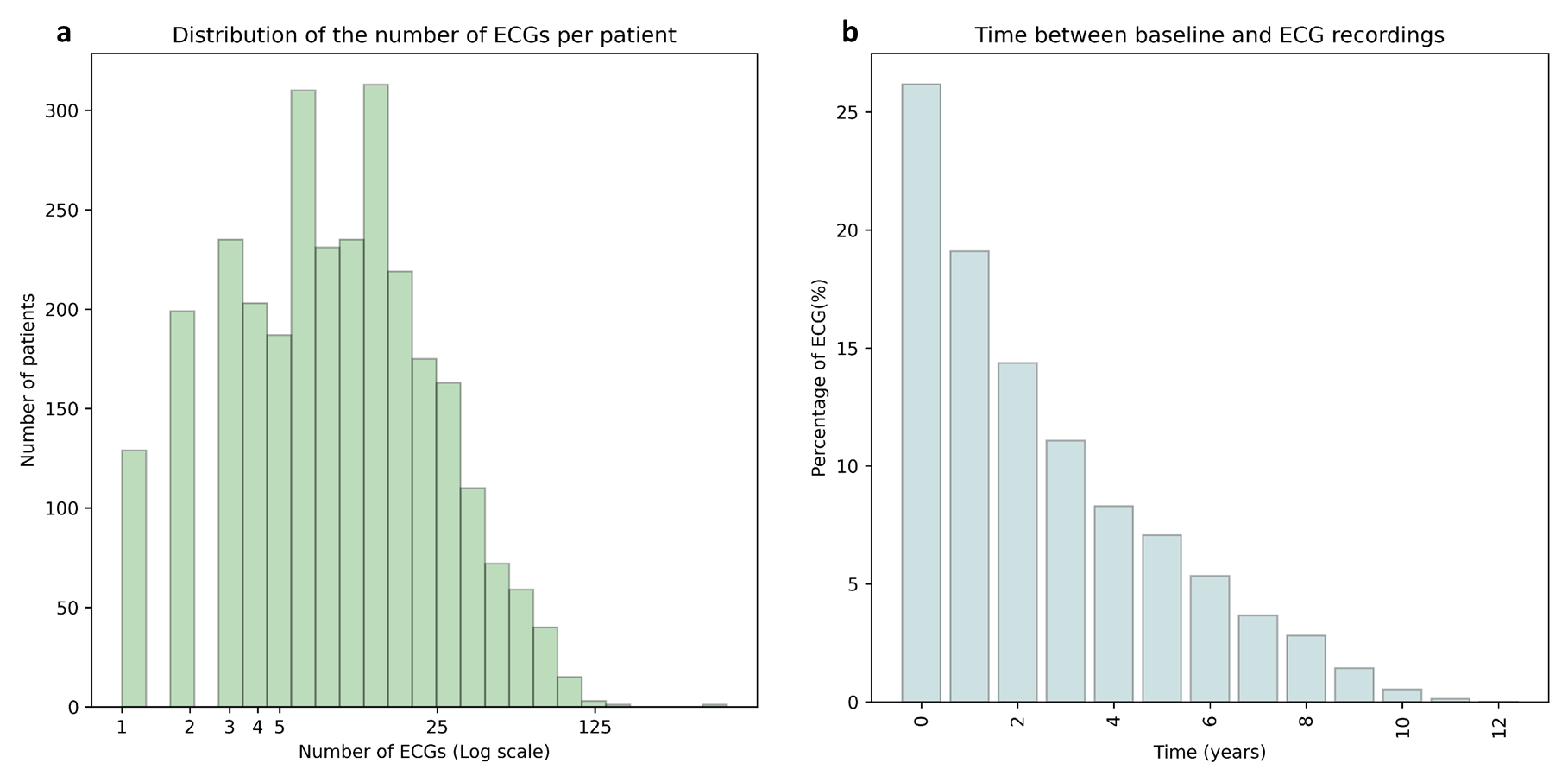
**

**Supplementary Figure 3. Schematic overview of variational autoencoder (VAE) architecture components. The central component of the VAE is represented by the 3 fully connected layers, which are essential for the encoding and decoding processes within the network. In the encoding phase, they transform input data into a latent space representation (a mean and a standard deviation latent parameter layers, which parametrize the Gaussian distribution of the latent space), capturing essential features and reducing the dimensionality of the data. In the decoding phase, these layers reconstruct the input data from the latent distributions (Z), generating output that closely resembles the original input.**

**
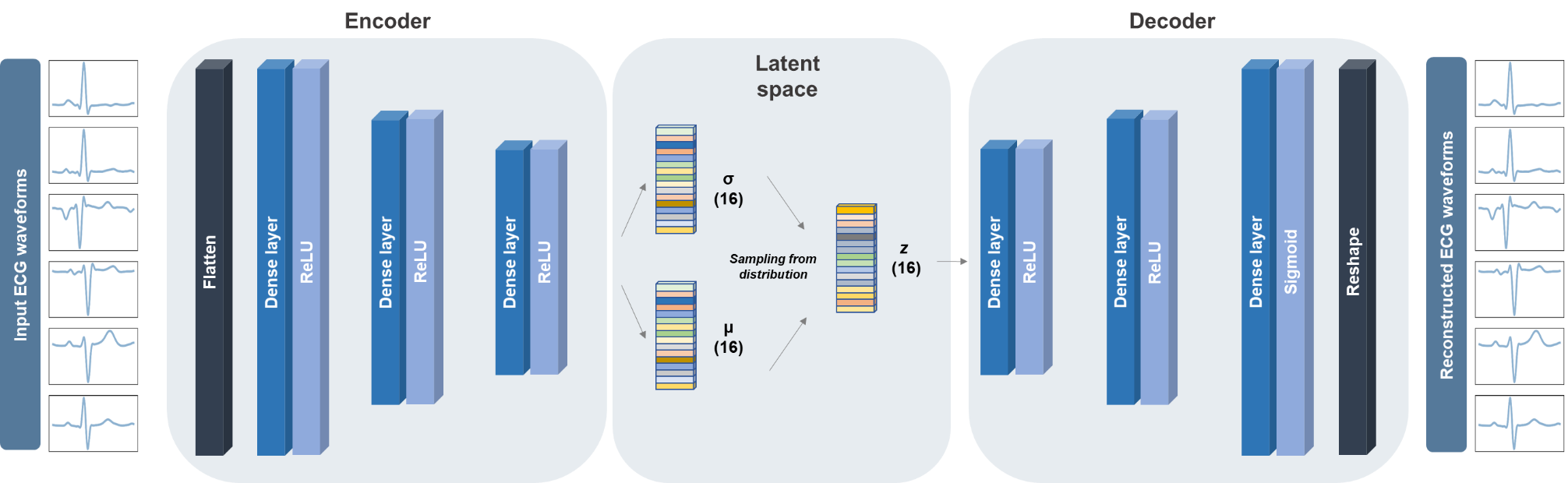
 Supplementary Figure 4. Survival curves for the outcomes of interest**


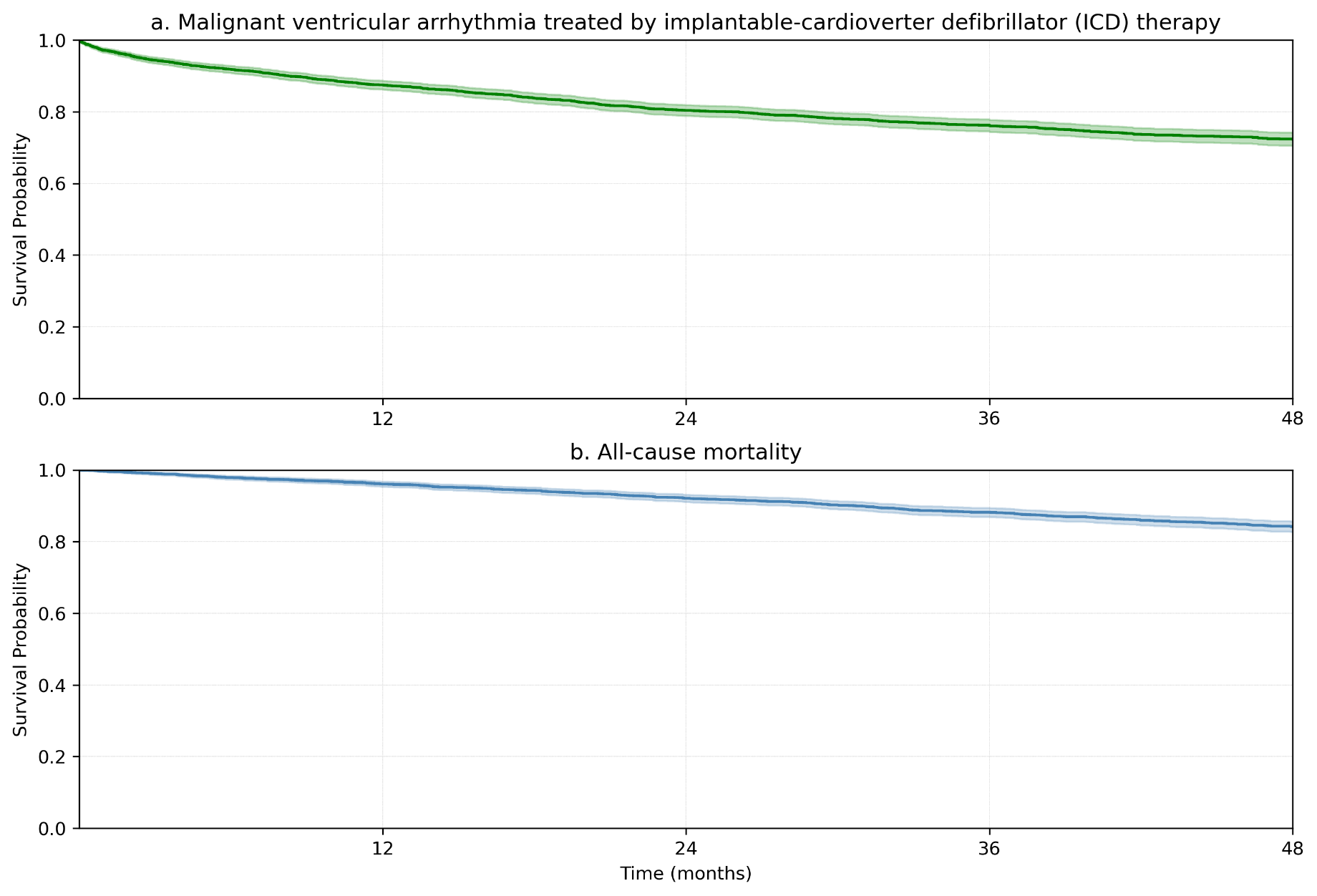


**Supplementary figure 5. Example of the original 6-lead ECG (blue) and a reconstructed signal (orange) using the variational autoencoder (VAE)**


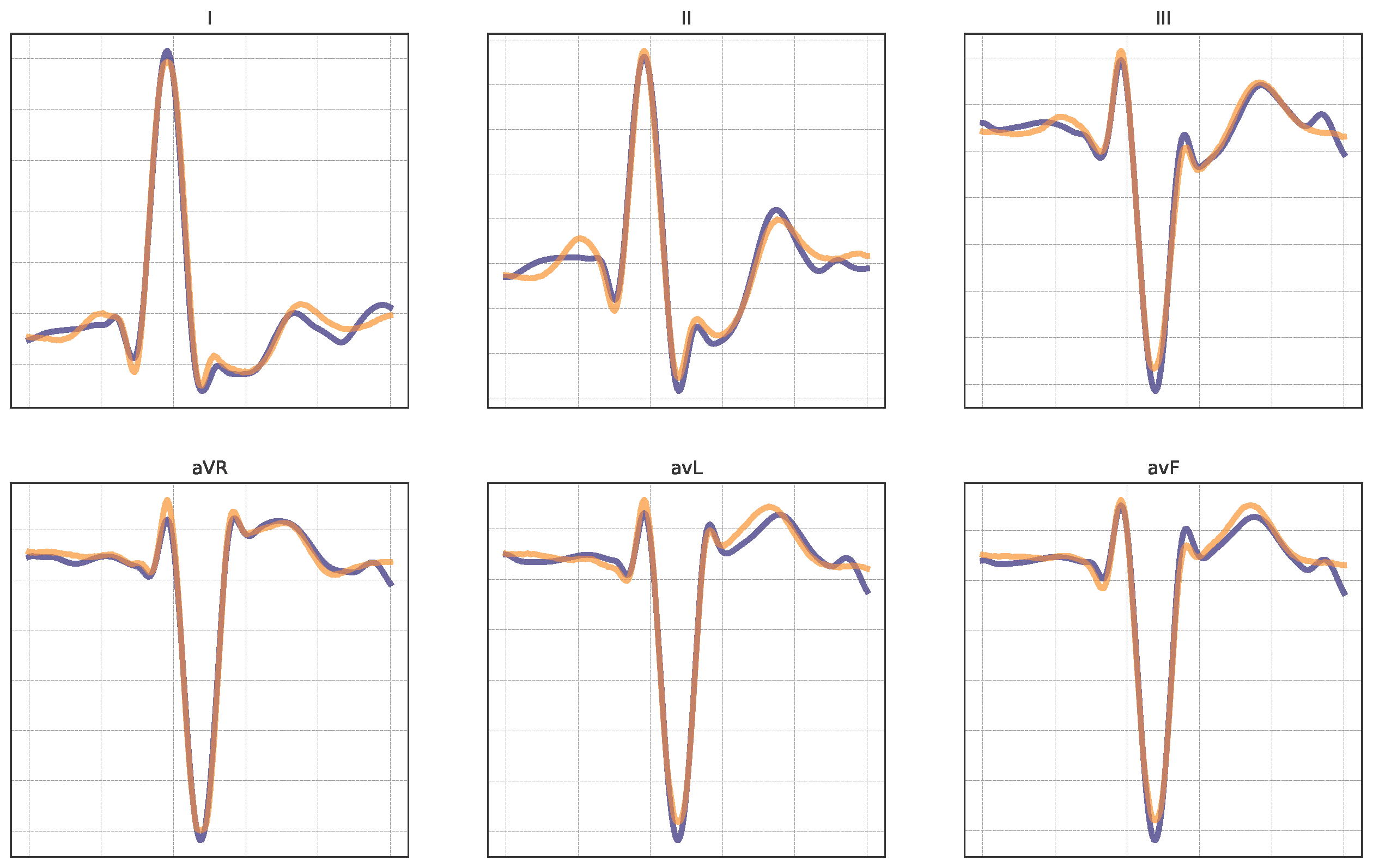


**Supplementary Figure 6. Calibration plot of the dynamic model**





**Supplementary Figure 7. Time-dependent area under the curve (AUROC) for the 6-lead and the single-lead dynamic models**


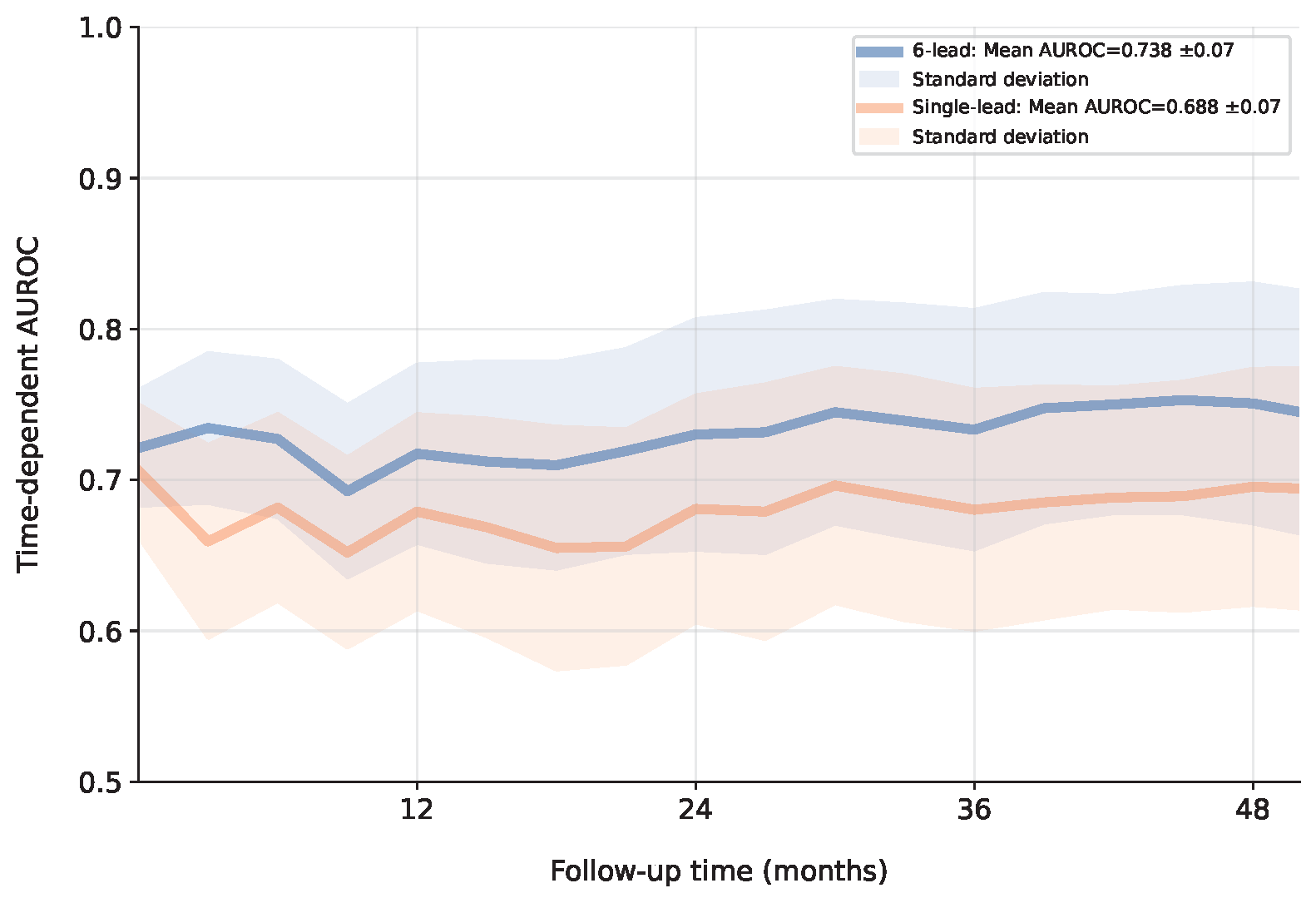


**Supplementary Figure 8.** Time-dependent area under the curve (AUROC) for the dynamic models trained on VAE-derived latent space variables or clinical ECG interpretations


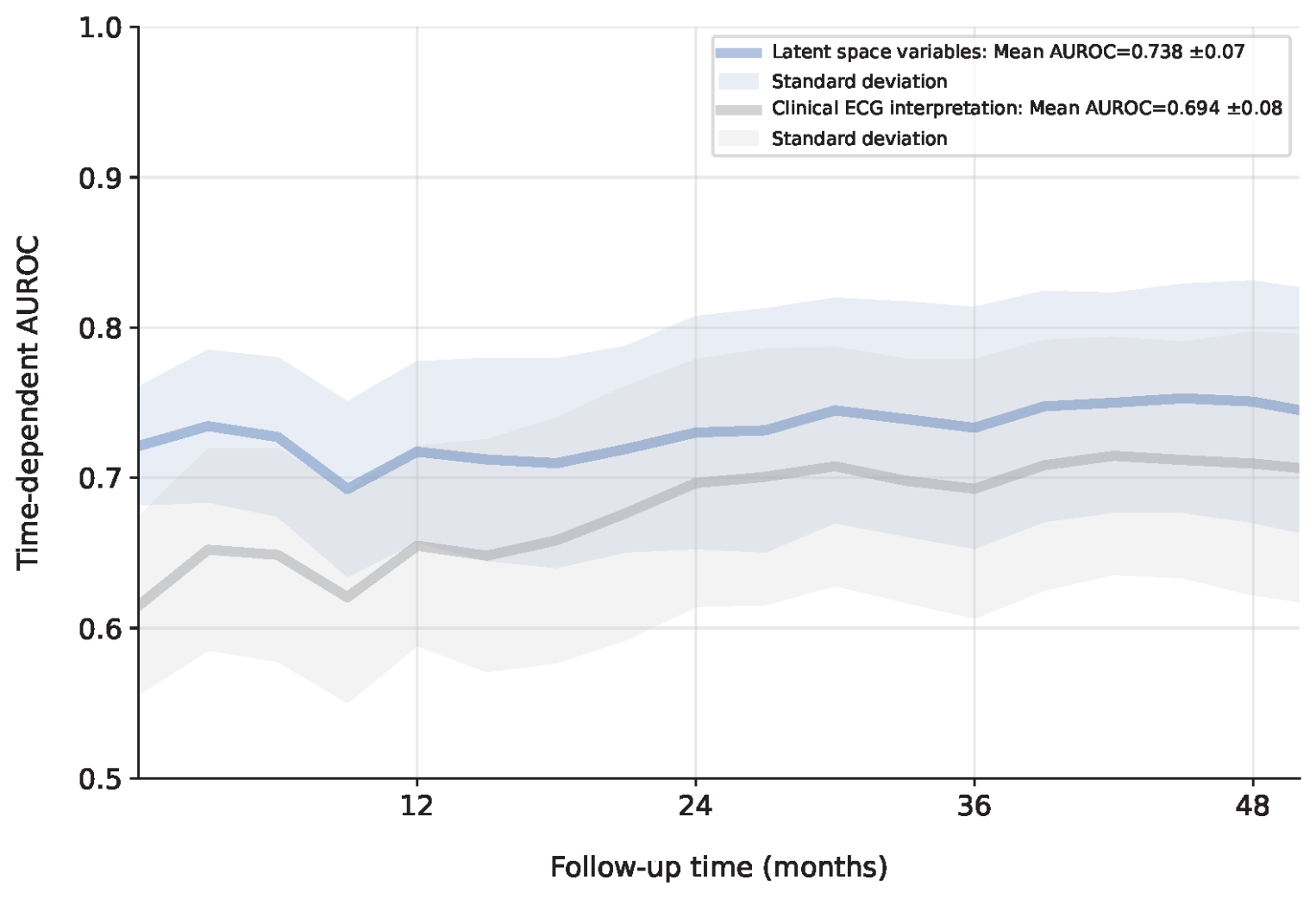


**Supplementary Figure 9.** Time-dependent area under the curve (AUROC) for the prediction of all-cause mortality compared to malignant ventricular arrhythmias


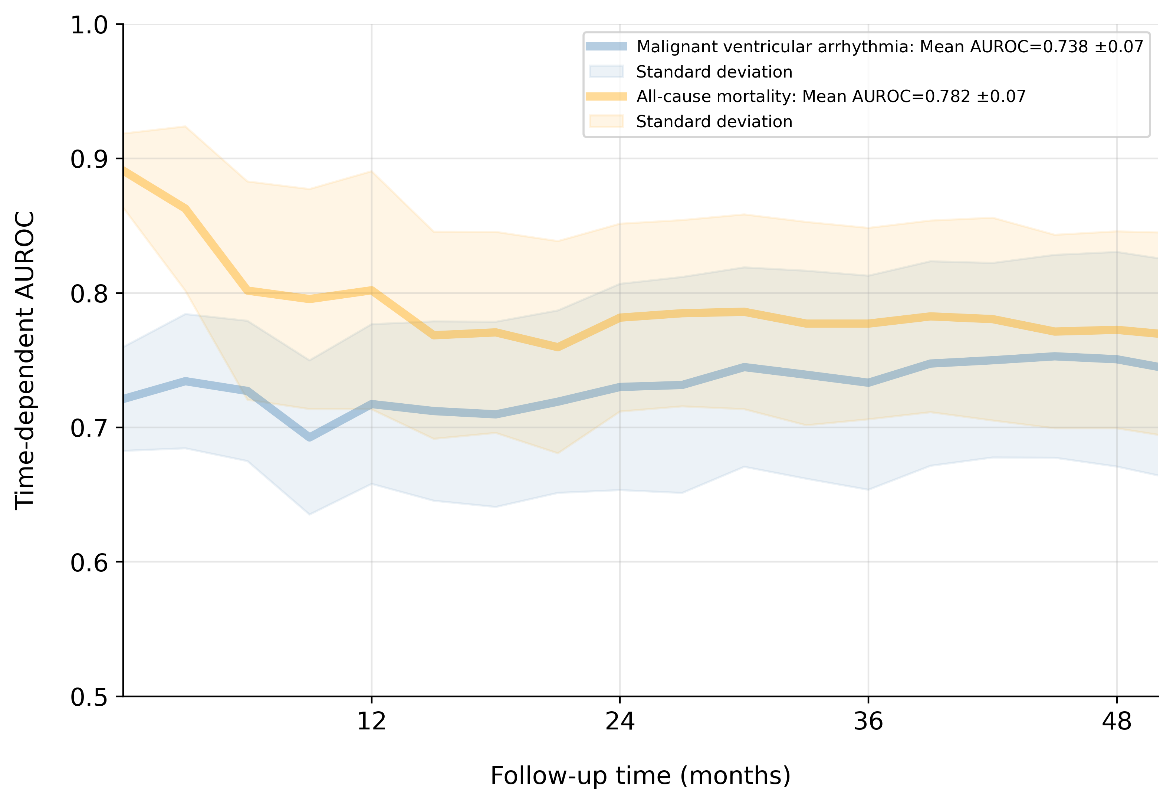


**Supplementary Figure 10.** Time-dependent area under the curve (AUROC) for the prediction malignant ventricular arrhythmias treated by shock and/or anti-tachycardia pacing (ATP) versus shock-only
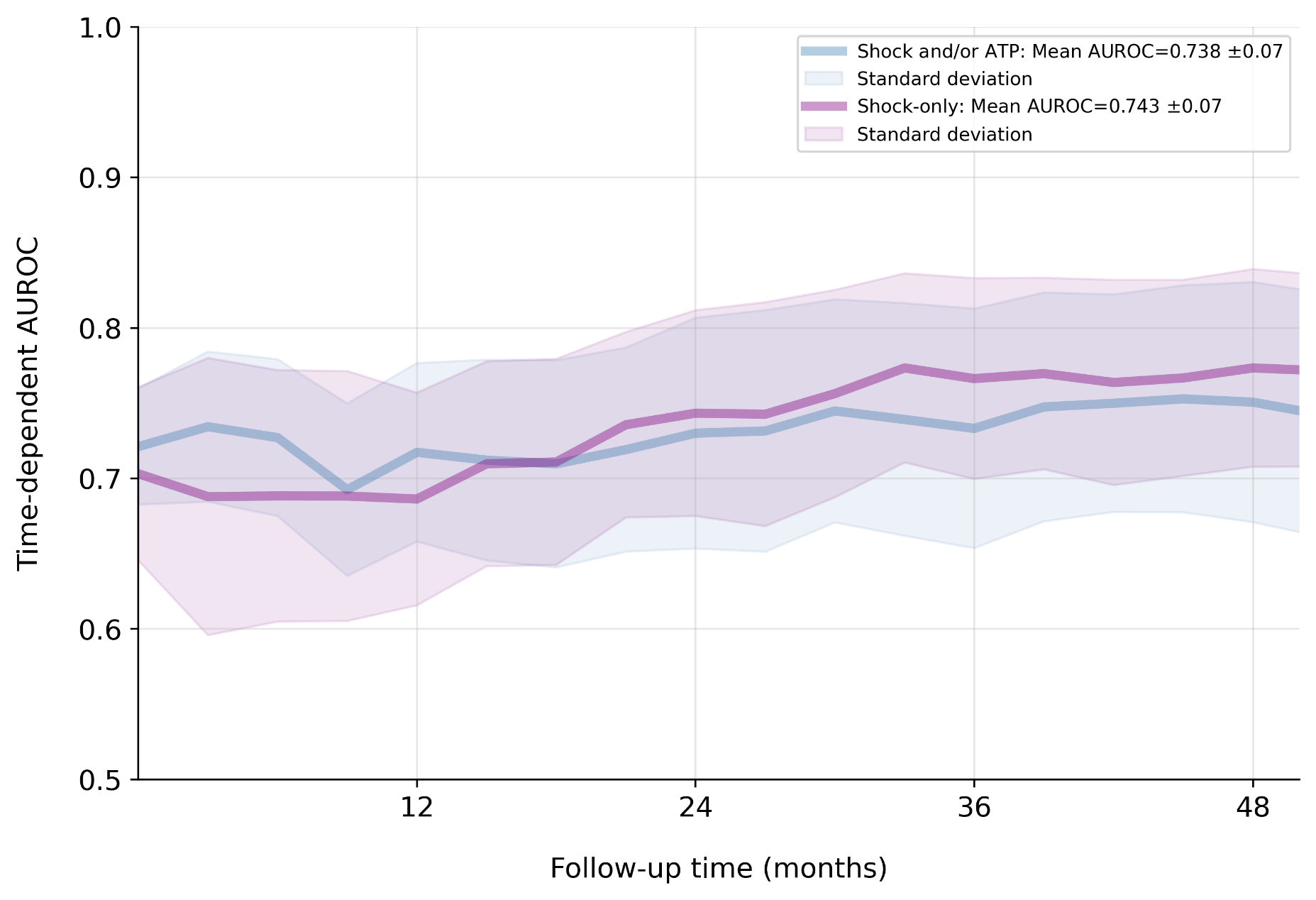


**Supplementary Figure 11. Validation of model performance in patient subgroups
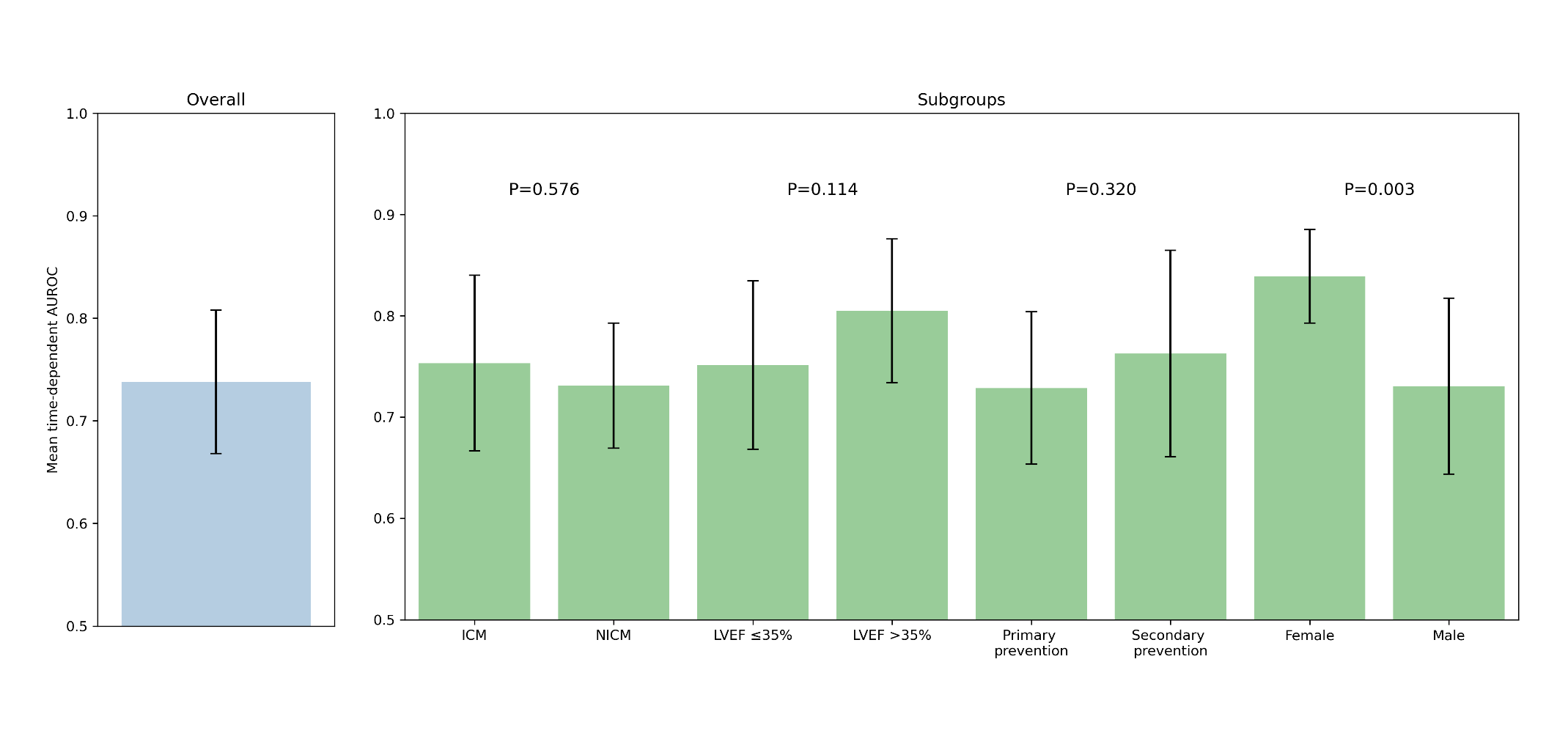
**

**Supplementary Figure 12. Factor traverse for each latent space variable
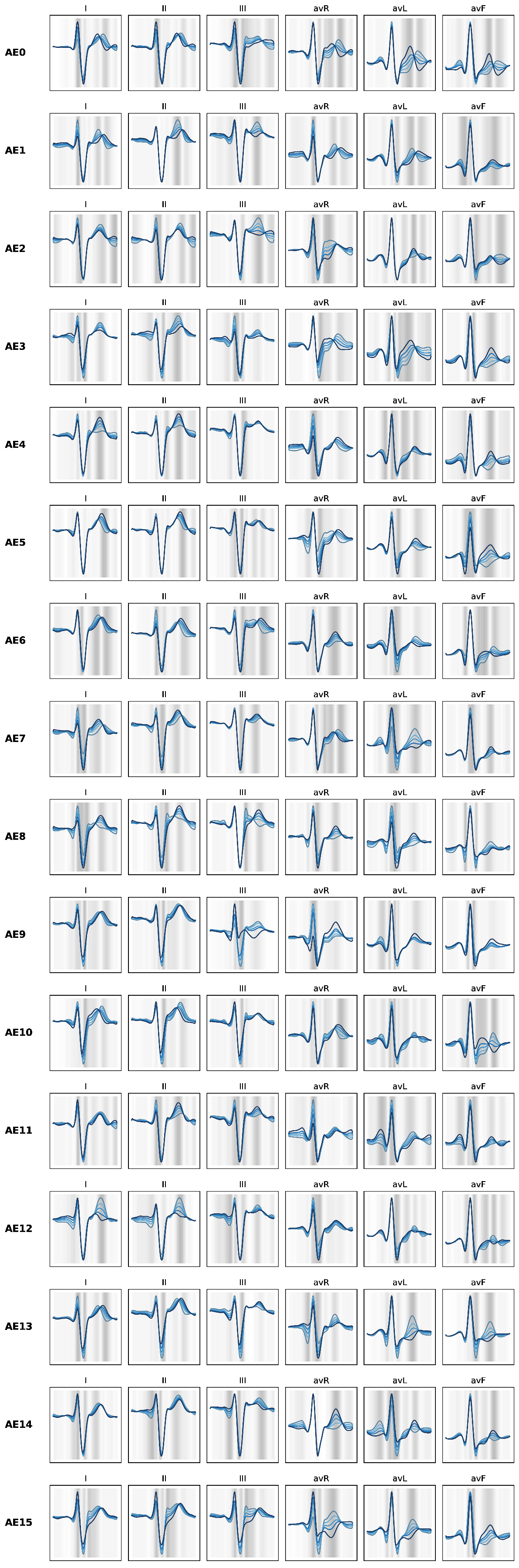
**

**
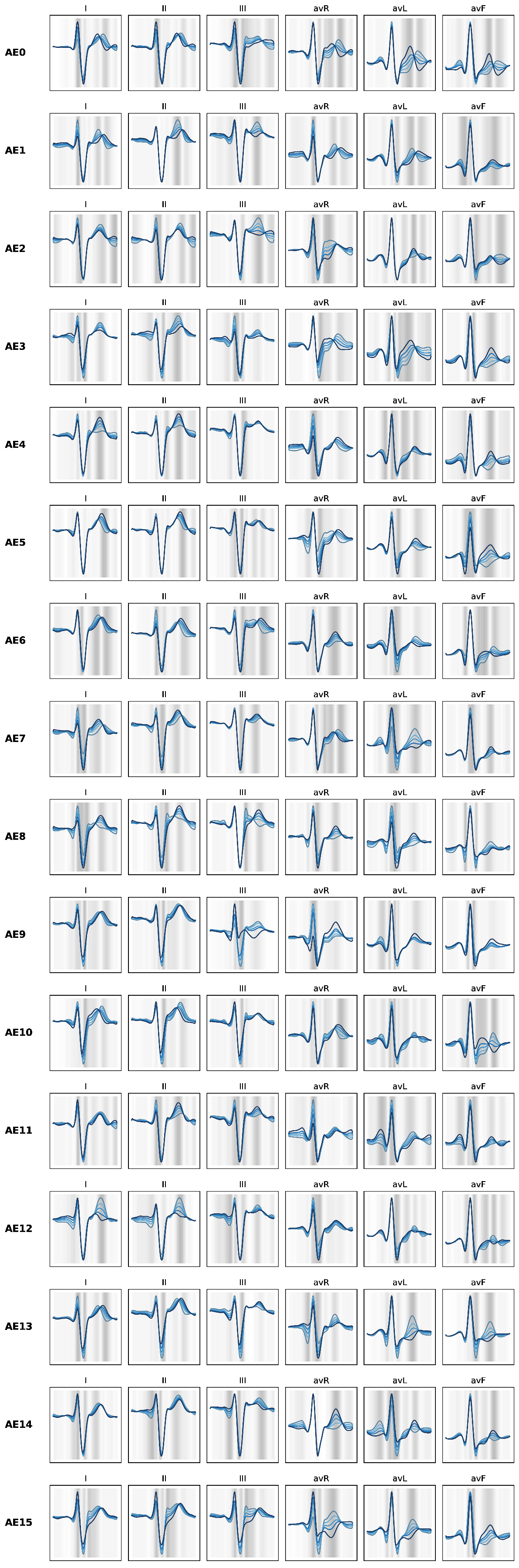
**

**Supplementary Figure 13. Heatmap showing the correlations between the latent space variables and the human-interpretable ECG interpretations and measurements**
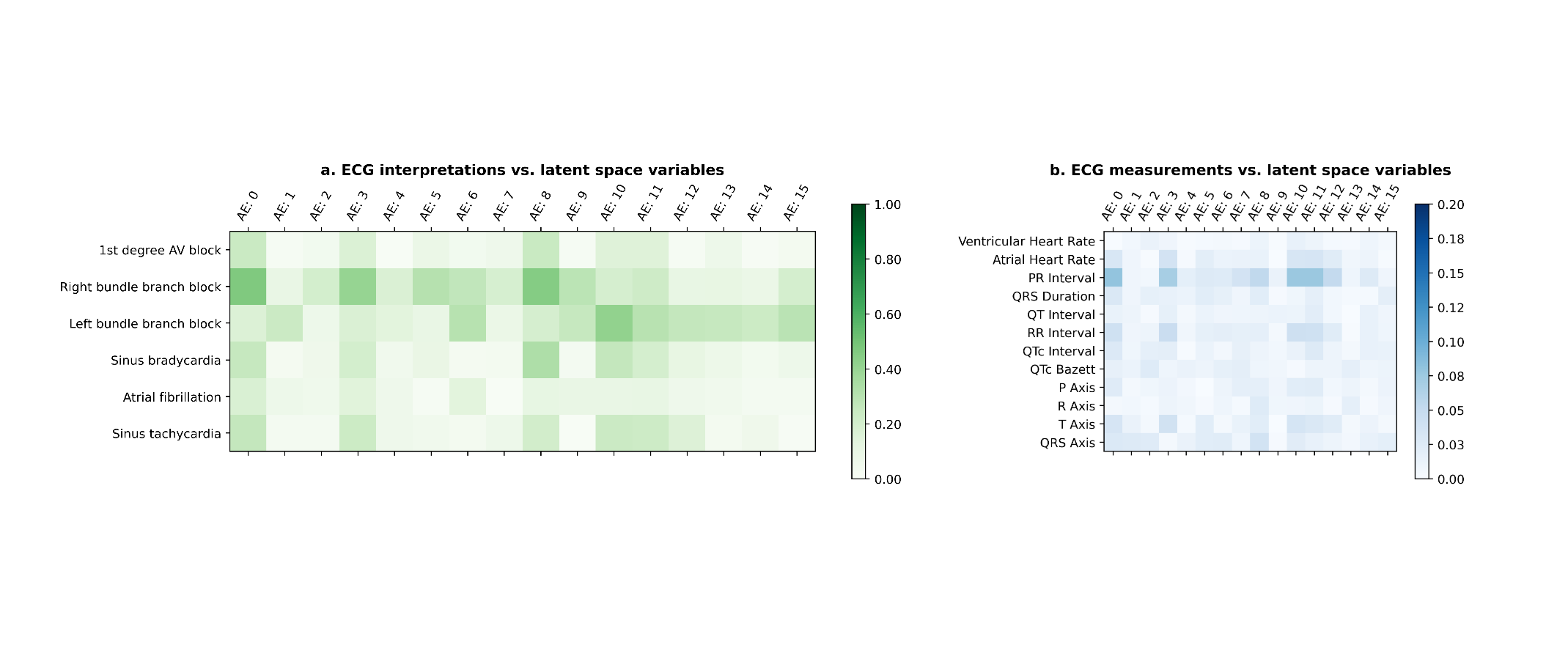


**References**

1. Beetz M, Banerjee A, Grau V. Multi-Domain Variational Autoencoders for Combined Modeling of MRI-Based Biventricular Anatomy and ECG-Based Cardiac Electrophysiology. *Front Physiol* 2022; **13**: 886723.

2. Zhu F, Ye F, Fu Y, Liu Q, Shen B. Electrocardiogram generation with a bidirectional LSTM-CNN generative adversarial network. *Sci Rep* 2019; **9**(1): 6734.

3. van de Leur RR, Bos MN, Taha K, et al. Improving explainability of deep neural network-based electrocardiogram interpretation using variational auto-encoders(). *Eur Heart J Digit Health* 2022; **3**(3): 390-404.

4. Delaney AM BE, Ward TE. Synthesis of Realistic ECG using Generative Adversarial Networks. *arXiv* 2019; **1909**.
